# Effect of Polyhexamethylene Biguanide in Combination with Undecylenamidopropyl Betaine or PslG on Biofilm Clearance

**DOI:** 10.3390/ijms22020768

**Published:** 2021-01-14

**Authors:** Yaqian Zheng, Di Wang, Luyan Z. Ma

**Affiliations:** 1 State Key Laboratory of Microbial Resources, Institute of Microbiology, Chinese Academy of Sciences, Beijing 100101, China; zhengyaqian18@mails.ucas.ac.cn (Y.Z.); wangdi@im.ac.cn (D.W.); 2 College of Life Sciences, University of Chinese Academy of Sciences, Beijing 100049, China; 3 Savaid Medical School, University of Chinese Academy of Sciences, Beijing 100049, China

**Keywords:** biofilm, polyhexamethylene biguanide (PHMB), undecylenamidopropyl betaine (UB), PslG

## Abstract

Hospital-acquired infection is a great challenge for clinical treatment due to pathogens’ biofilm formation and their antibiotic resistance. Here, we investigate the effect of antiseptic agent polyhexamethylene biguanide (PHMB) and undecylenamidopropyl betaine (UB) against biofilms of four pathogens that are often found in hospitals, including Gram-negative bacteria *Pseudomonas aeruginosa* and *Escherichia coli*, Gram-positive bacteria *Staphylococcus aureus*, and pathogenic fungus, *Candida albicans*. We show that 0.02% PHMB, which is 10-fold lower than the concentration of commercial products, has a strong inhibitory effect on the growth, initial attachment, and biofilm formation of all tested pathogens. PHMB can also disrupt the preformed biofilms of these pathogens. In contrast, 0.1% UB exhibits a mild inhibitory effect on biofilm formation of the four pathogens. This concentration inhibits the growth of *S*. *aureus* and *C. albicans* yet has no growth effect on *P. aeruginosa* or *E. coli*. UB only slightly enhances the anti-biofilm efficacy of PHMB on *P. aeruginosa* biofilms. However, pretreatment with PslG, a glycosyl hydrolase that can efficiently inhibit and disrupt *P. aeruginosa* biofilm, highly enhances the clearance effect of PHMB on *P. aeruginosa* biofilms. Meanwhile, PslG can also disassemble the preformed biofilms of the other three pathogens within 30 min to a similar extent as UB treatment for 24 h.

## 1. Introduction

High-risk pathogens associated with nosocomial infections often disseminate rapidly through the hospital environment, and are threats to the health of patients, especially those with comprised immune systems. Under the threat of the same disease, in-hospital mortality was much higher in patients with nosocomial infections than those without [1]. The most common pathogens that cause nosocomial infections including Gram-negative bacteria *Pseudomonas aeruginosa* and *Escherichia coli*, Gram-positive bacteria *Staphylococcus aureus*, and pathogenic fungus *Candida albicans*, often tend to form biofilms, which are highly resistant to clearance and antibiotic treatment [2,3].

Biofilms are communities of microorganisms embedded in an extracellular polymeric substances (EPS) matrix. Biofilm microbes exhibit extreme tolerance to antibiotics and are protected from the host immune clearance [4]. Development of biofilm generally includes an attachment stage, a biofilm maturation stage, and a biofilm dispersion stage [5,6]. Microbial cells secreted extracellular substances and formed biofilm matrix, which plays a key role in biofilm development [7]. In general, biofilm matrix is composed of extracellular polysaccharides, proteins, and extracellular DNA [8]. Multiple strategies have been developed targeting these matrix components to eradicate microbial biofilms, such as treatments with DNase I or Proteinase [9,10,11]. Our previous studies have shown that PslG, an endoglycosidase, is capable of degrading exopolysaccharide Psl, a critical biofilm matrix component of *P. aeruginosa*, leading to prevention of biofilm formation and disassembly of existing biofilms [12,13]. PslG alone does not kill bacterial cells. However, it has been proved that PslG treatment could sensitize biofilm bacteria to antibiotics and improve eradication of biofilms [12]. It is not clear whether PslG treatment could also enhance the bactericidal efficacy of other antimicrobial therapies, such as antiseptics. 

Regarding the increased antibiotic resistance caused by biofilms, antiseptics are required to inhibit the pathogen growth, remove the biofilms, and control the nosocomial infections [14]. Polyhexanide (polyhexamethylene biguanide, PHMB), a biocide of the bisbiguanide family, is one of these antiseptics with bactericidal activity at very low concentrations (0.1%) [15]. It is a cationic polymer and capable of attaching to negatively charged membrane lipids (including Gram-negative and Gram-positive membranes), leading to membrane permeability [16,17]. PHMB has been reported to have great antimicrobial activity against both gram-negative and gram-positive bacteria, and also effective against pathogenic fungus, for instance, *C. albicans* [18,19]. Meanwhile, PHMB has minor effects on human membranes and had very good tissue compatibility [14,20,21]. It has been widely used as a surface disinfectant and also applied in clinical practice, for example, in wound treatment and promoting wound healing [22,23]. Commercial disinfectants and antiseptic products often add betaine in PHMB solutions [18]. Undecylenamidopropyl betaine (UB) is a surfactant with antimicrobial properties, which has been proved to be capable of reducing cytotoxicity of PHMB and increasing the antimicrobial activity of PHMB [24,25]. Previous studies have discovered that the combined PHMB with UB could effectively remove the biofilms formed by pathogens, such as *S. aureus* and *P. aeruginosa* [26]. However, it is not clear which of these two components exhibited the main antimicrobial function and how the combined solution worked at different stages of biofilms. Antiseptics should not be toxic to the host. The concentration of PHMB as antiseptic was commonly between 0.1~0.2% [27,28,29]. However, considering the production cost and toxicity, the lower dose, the better. Thus, the lower dose of PHMB to inhibit the high-risk pathogens’ growth, biofilm formation, as well as to disrupt preformed biofilms is also determined in this study. 

In our present study, we selected four high-risk pathogens associated with nosocomial infections to test the anti-biofilm ability of antiseptic agents against bacteria, including gram-negative bacteria *P. aeruginosa* and *E. coli*, gram-positive bacteria *S. aureus*, and pathogenic fungus, *C. albicans*. All these four pathogens are the common pathogens in clinical settings that cause both acute and chronic infections [30,31]. With biofilm formation, these pathogens often exhibited serious antibiotic resistance and are notoriously difficult to eradicate [2,32,33,34]. We investigated the effect of PHMB and UB on pathogens biofilms by using them separately or in combination. In addition, we also tested whether PslG had a synergistic action with PHMB against the pathogens’ preformed biofilms. Pretreatment with PslG strongly enhanced the efficiency of PHMB at disrupting *P. aeruginosa* preformed biofilms, suggesting a great prospect of PslG in antiseptic development.

## 2. Results

### 2.1. The Concentration of PHMB that Can Prevent Biofilm Formation and Disrupt the Preformed Biofilm of P. aeruginosa

**Figure 1:**
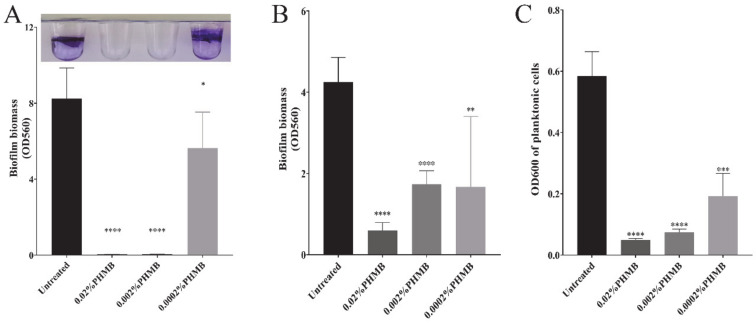
Determination of the polyhexamethylene biguanide (PHMB) concentration that can prevent biofilm formation and disrupt the preformed biofilm of *P. aeruginosa*. PHMB with concentrations from 0.02% to 0.0002% was added to *P. aeruginosa* biofilm culture to determine: (**A**) The inhibitory concentration of PHMB on *P. aeruginosa* biofilm formation. (**B**) PHMB concentration on removing *P. aeruginosa* preformed biofilms. The culture of 24-h-old *P. aeruginosa* biofilms were replaced with either fresh Jensen’s medium (Untreated column) or a series of concentrations of PHMB diluted in Jensen’s medium. After 24 h of treatment, the remaining biofilm biomass on wells were determined. (**C**) Bactericidal concentration of PHMB at killing planktonic cells in preformed biofilm culture of *P. aeruginosa*. After 24 h of treatment, optical density at 600 nm (OD _600_) of planktonic cells in each well was measured. Shown are the averages of three independent experiments and standard deviations. Significance were determined using Student’s *t*-test (* *p* < 0.05; ** *p* < 0.01; *** *p* < 0.001; **** *p* < 0.0001).

In most commercial disinfectants, PHMB concentration is used at 0.1~0.2% [28,29]. Harbs N et al. have reported that a lower concentration of PHMB (0.02%) is able to remove the *P. aeruginosa* biofilms. However, it is not clear whether this concentration or even lower concentrations would be able to inhibit the pathogen’s growth or control biofilm formation. Here, we determined the concentration of PHMB that can prevent biofilm formation and disrupt the preformed biofilm of *P. aeruginosa*. PHMB was diluted to concentrations at 0.02%, 0.002% and 0.0002%. Each concentration of PHMB was added to *P. aeruginosa* growth culture. PHMB effect on bacteria biofilms was determined by crystal violet staining assay. PHMB at 0.02% and 0.002% could effectively prevent the biofilm formation of *P. aeruginosa* (Figure 1A). However, 0.002% PHMB could only disrupt the biofilm by 50% when applied to a 24 h-old biofilm, which is usually considered to be a mature biofilm (Figure 1B). PHMB at 0.02% remarkably disrupted the 24 h-old biofilm by over 80% (Figure 1B), and the treated culture had very low OD_600_ (Figure 1C), suggesting that most biofilm bacteria were dead. Thus, 0.02% PHMB is the concentration that is able to prevent and eradicate *P. aeruginosa* biofilms. In the following experiment, we stayed on 0.02% of PHMB, unless specifically indicated.

### 2.2. Effect of PHMB, UB, and PHMB + UB on Different Stages of P. aeruginosa Biofilm Development

**Figure 2:**
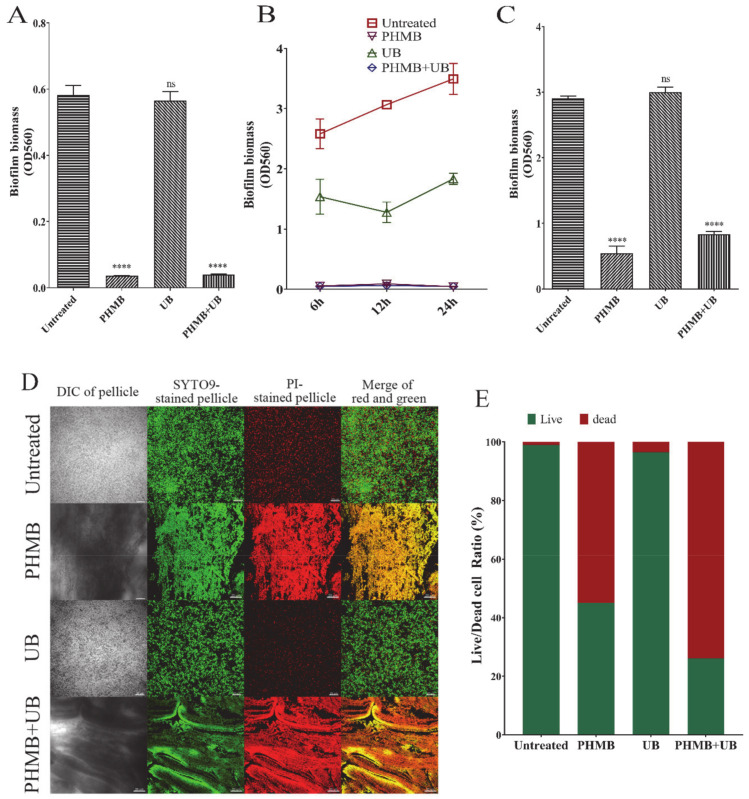
Inhibitory effect of PHMB, undecylenamidopropyl betaine (UB), and PHMB + UB (PHMB in combination with UB) on different stages of *P. aeruginosa* biofilms. 0.02% PHMB and 0.1% UB was applied to determine their individual or in combination effect on (**A**) preventing *P. aeruginosa* attachment; (**B**) inhibiting biofilm formation from early to late stages; (**C**) disrupting preformed biofilm in 24 h. (**D**) Live/Dead staining of *P. aeruginosa* biofilm was applied to determine the bactericidal capacity of PHMB and UB on *P. aeruginosa* biofilm cells. *P. aeruginosa* 24 h-old pellicles were treated with PHMB, UB, and PHMB+UB for 6 h. Images were acquired by confocal laser scanning microscopy (CLSM). Live bacteria were stained green with SYTO 9. Dead bacteria were stained red with propidium iodide (PI) [19]. Scale bar, 20 μm. (**E**) Live/dead-cell ratio of *P. aeruginosa* pellicles with PHMB, UB, and PHMB+UB treatments for 6h. Biofilm biomass was quantified using COMSTAT software. Significance were determined using Student ’s *t*-test (“ns”, not significant, *p* > 0.05; **** *p* < 0.0001).

Commercial disinfectant and antiseptic products commonly contained PHMB and UB. In order to distinguish the individual effect of PHMB and UB on biofilms, we examined the effect of PHMB, UB, and PHMB + UB (PHMB in combination with UB) on *P. aeruginosa* biofilm, respectively. We first tested their effect on biofilm initial attachment. PHMB (0.02%) reduced the *P. aeruginosa* attachment by ~90%, while 0.1% UB hardly exhibited any effect (Figure 2A). The combination of PHMB and UB did not improve the inhibitory effect of PHMB, indicating that UB is not effective at preventing bacterial attachment. We then detected the effect of PHMB and UB on biofilm formation at three different time points (6 h, 12 h, and 24 h-growth). At all these three time points, PHMB completely inhibited *P. aeruginosa* biofilm formation. UB showed a partial inhibitory effect, by reducing the biofilm biomass by around 50% (Figure 2B). However, when applied to preformed *P. aeruginosa* biofilms, UB had no effect on maturely formed biofilms, yet PHMB reduced 24 h-biofilms by near 75%, compared to untreated control (Figure 2C). These results suggested that 0.02% PHMB was efficient at preventing all the stages of *P. aeruginosa* biofilm development. UB only had a slight inhibitory effect on biofilm formation, but was not efficient enough at preventing attachment, nor disrupting preformed biofilms. 

**Figure 3:**
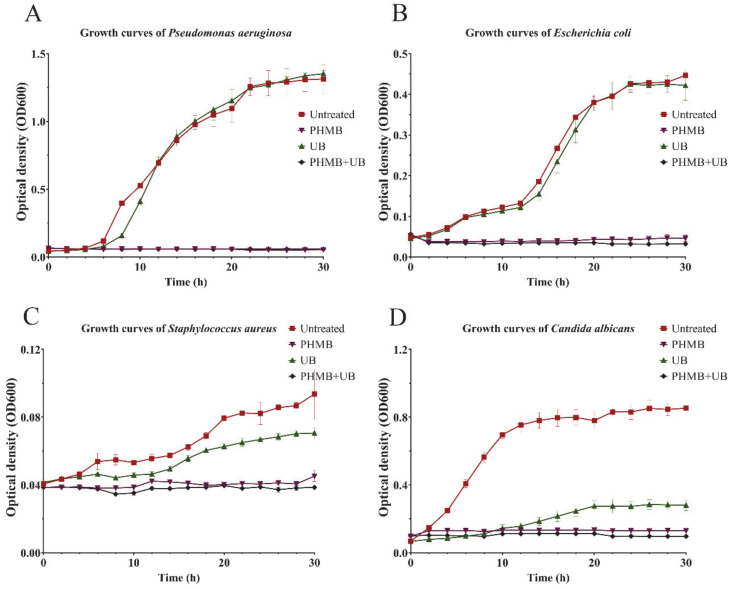
Inhibitory effect of PHMB, UB, and PHMB in combination with UB (PHMB + UB) on the growth of high-risk pathogens. Figures represent the growth curves of (**A**) *P. aeruginosa*; (**B**) *E. coli*; (**C**) *S. aureus*; (**D**) *C. albicans* with different antiseptics treatments. Each experiment was performed with triplicates.

We then compared the bactericidal capacity of PHMB and UB to check if that was the reason causing the differences between PHMB and UB at preventing *P. aeruginosa* biofilm formation. With the presence of 0.02% PHMB, *P. aeruginosa* could not grow at all, while 0.1% UB alone did not had any killing effect on *P. aeruginosa* (Figure 3A). Live/Dead staining on *P. aeruginosa* biofilms after 6 h treatment also indicated that UB had no significant killing effect on biofilm bacteria, yet PHMB killed 60% bacteria within biofilms (Figure 2D,E). However, PHMB + UB had over 75% dead bacteria, suggesting UB might slightly enhance the killing of PHMB on biofilm bacteria of *P. aeruginosa* (Figure 2D,E).

### 2.3. PHMB and UB against Biofilms Formed by Different Types of Pathogens

**Figure 4:**
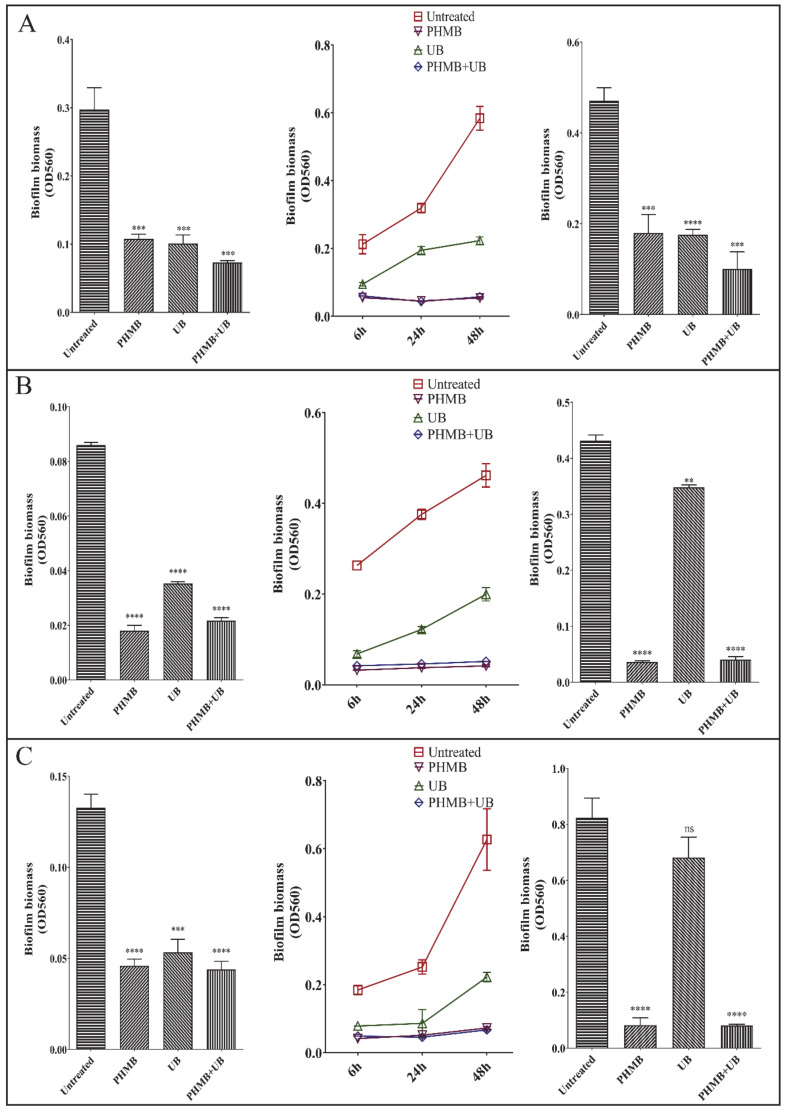
Bactericidal capacity of PHMB, UB, and PHMB + UB against biofilms of different pathogens: (**A**) *E. coli*; (**B**) *S. aureus*; (**C**) *C. albicans*. Left panels represent the effect of antiseptics on preventing pathogens attachment. Middle panels show the effect of antiseptics at inhibiting biofilm formation. Right panels indicate clearance capacity of antiseptics on preformed biofilms of each pathogen. Shown are the averages of three independent experiments and standard deviations. Significance were determined using Student ’s *t*-test (“ns”, not significant, *p* > 0.05; ** *p* < 0.01; *** *p* < 0.001; **** *p* < 0.0001).

We then determined the bactericidal capacity of 0.02% PHMB and 0.1% UB on three other pathogens, including gram-negative pathogen *E. coli*, gram-positive pathogen *S. aureus*, and *C. albicans*, the main pathogen of nosocomial fungal infections. PHMB and UB exhibited different antimicrobial activities on different pathogens. PHMB could completely prevent all of these three pathogens growth in 30 h, while UB had no effect on *E. coli* growth, which is similar to the result of another gram-negative pathogen, *P. aeruginosa.* Nevertheless, UB inhibited *S. aureus* growth by 50%, and reduced *C. albicans* growth to 90% (Figure 3). In consistent with the bactericidal ability, 0.02% PHMB and 0.1% UB were effective at preventing the biofilm formation of these three pathogens (Figure 4, Left and middle panels). However, the combination of PHMB with UB did not improve the capacity of PHMB against the biofilms of three pathogens. When applied to preformed biofilms, PHMB could disrupt all these four pathogens’ biofilms, while UB was only efficient for *E. coli* biofilms (Figure 4, right panels). Considering the fact that UB did not inhibit the growth of *E. coli*, the eradication effect of UB on *E. coli* biofilm might not due to its bactericidal activity. 

### 2.4. Combination of PHMB and PslG Enhanced Biofilms Clearance

**Figure 5:**
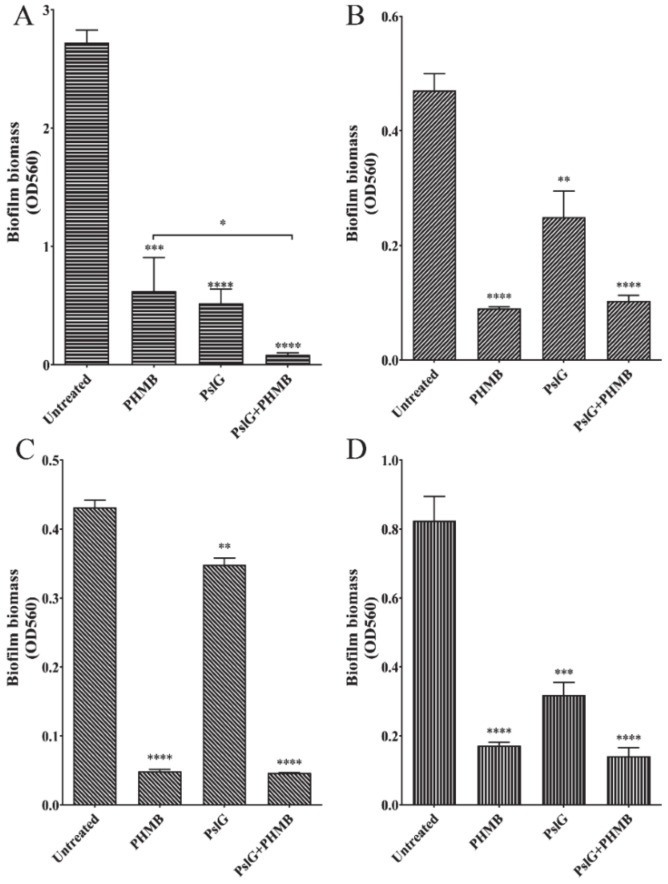
PslG treatment efficiently reduced the biofilm biomass of different pathogens. Preformed biofilms were treated with PHMB for 2 h, PslG for 30 min, or PHMB (2 h) after pre-treatment with PslG for 30 min (PslG+PHMB). (**A**) Disruption of *P. aeruginosa* biofilm. (**B**) Disruption of *E. coli* biofilm. (**C**) Disruption of *S. aureus* biofilm. (**D**) Disruption of *C. albicans* biofilm. Shown are the averages of three independent experiments and standard deviations. Significance were determined using Student ’s *t*-test (* *p* < 0.05; ** *p* < 0.01; *** *p* < 0.001; **** *p* < 0.0001).

PslG is a self-produced glycosyl hydrolase of *P. aeruginosa* that has been proved to be able to disrupt exopolysaccharide matrix in biofilm and trigger biofilm disassembly in *P. aeruginosa*. However, it does not kill bacterial cells [12]. Here we applied PHMB in combination of PslG on biofilms, to check if these two treatments together could generate even stronger ability of clearance for *P. aeruginosa* biofilm. Since PHMB can interfere with the surface charge of protein, we treated the 24 h-old biofilms with PslG (50 nM) for 30 min firstly, and then added 0.02% PHMB to the culture to grow *P. aeruginosa* biofilm. Two hours later, biofilm biomass was determined by crystal violet staining assay. PHMB and PslG alone could reduce the biofilm biomass by 40% and 60%, respectively (Figure 5A). Pretreatment with PslG enhanced the PHMB efficacy of eradicating biofilms, and in combination, they reduced 80% of biofilm biomass. Presumably, PslG pretreatment disrupted the biofilm matrix, which allowed PHMB to get into deeper layers of biofilms to engage with microbial cells within biofilms and enhanced the antimicrobial effect of PHMB. PslG, as a glycosyl hydrolase, only targeted on special substrates. As previously reported, PslG might cleave the bond between beta-D-mannose and alpha-L-Rhamnose of exopolysaccharide Psl in *P. aeruginosa* biofilm matrix. Surprisingly, we found that PslG also exhibited eradication effect on *E. coli* and *C. albicans* preformed biofilms (Figure 5B,D), indicating that PslG might have a broad anti-biofilm activity against high-risk pathogen biofilms.

## 3. Discussion

PHMB, as an efficient antiseptic, has been extensively studied for its bactericidal capacity on high-risk pathogens that cause nosocomial infections. Concentration of PHMB varied in a large range (from 0.1% to 20%) in the current commercial products [18,35]. Mulder GD et al. has reported that a wound dressing with 0.3% PHMB did not result in cytotoxicity [36]. However, a review by A. Kramer et al. has discussed the safety of PHMB and stated that PHMB at a concentration ≥0.025% showed cytotoxic effect on the peritoneum in guinea pigs [20]. Thus, finding out the minimal concentration of PHMB against most common pathogens would safely guide PHMB application on diverse foci of infections on different parts of the body. Here, we selected *P. aeruginosa* as a model pathogen to determine the concentration of PHMB on inhibiting pathogen growth, cell attachment, biofilm formation, and removal of preformed biofilms, respectively. PHMB at concentration of 0.02% could accomplish all of the missions listed above, while lower than that could not (Figure 1). Thus, 0.02% concentration of PHMB was applied through the entire work in this study and could be recommended to be used in future applications in clinical settings for both effectiveness and safety concerns.

Most studies of PHMB efficacy were focusing on the bactericidal capacity on planktonic cells. However, biofilms formed by high-risk pathogens account for over 60% of human chronic infections in hospital settings. Compared to planktonic cells, pathogen biofilms are more resistant to antibiotics and difficult to eradicate. There are a few studies that have investigated the clearance capacity of PHMB on some specific pathogen biofilms. However, there has been a lack of detailed study on the mechanism of PHMB on each step of biofilm formation, and whether there is any difference of the eradiation effect of PHMB on different types of high-risk pathogens biofilms. In our present work, we selected four high-risk pathogens as representatives of gram-negative, gram-positive bacteria and pathogenic fungus. We found that 0.02% PHMB could effectively prevent all these pathogens attachment (early stage of biofilm), inhibit their biofilm formation and disrupt their preformed biofilms (Figure 2 and Figure 4). CLSM observation confirmed that PHMB functioned not only at preventing the pathogen growth, but at killing bacteria cells (Figure 2). It is necessary to point out that in our CLSM result (Figure 2), pellicle samples with PHMB treatment had a distinctive appearance because the pellicles became extremely fragile after treatment and it was easy to cluster together.

Surfactant betaine (UB) has been added to PHMB into many antiseptic products due to its antimicrobial properties. However, its biocidal activity against different pathogen biofilms has not been clarified. In our study, we determined the effect of UB alone and UB in combination with PHMB against different pathogens’ biofilms, including cell growth, attachment, biofilm formation, and clearance of preformed biofilms. UB appeared to have no effect on gram-negative bacteria growth, including *P. aeruginosa* and *E. coli*, while having great antimicrobial effect on *S. aureus* and *C. albicans* (Figure 3). UB had a slight effect at inhibiting all these four pathogens biofilm formation. However, it could not prevent microbial attachment, nor disrupt preformed biofilms of *P. aeruginosa* (Figure 2 and Figure 4). The addition of UB also slightly enhanced the killing of biofilm bacteria of *P. aeruginosa* in this study (Figure 2E). This is consistent with the previous report [18]. In addition, UB had also been proved to be capable of reducing cytotoxicity of PHMB. Thus, the future work should carefully consider whether to add UB in the use of PHMB according to the type of pathogens and the foci of infections.

In our previous studies, we have reported PslG, a self-produced glycosyl hydrolase of *P. aeruginosa*, was capable of disassembling several *Pseudomonas* mature biofilms. However, PslG seemed to have little effect on the biofilms of *E. coli*, *S. aureus* and *C. albicans* [12]. Astonishingly, in this present work, we found that PslG actually is able to disassemble the preformed biofilms of these three pathogens, *E. coli*, *S. aureus*, and *C. albicans* (Figure 5). Treatment of PslG for 30 min had a similar or a better clearance activity as that of UB treatment for 24 h (Figure 2, Figure 4 and Figure 5). We compared these experiments performed in two studies and realized that the media used in these two studies were different. Considering the fact that PHMB could cause protein aggregation, we used minimal media to grow all the pathogen biofilms and determine the effect of antiseptics and PslG on biofilm clearance (Table 1). In contrast, biofilm formation in the precious work was performed in rich media to get a better and fast growth of pathogens. The media difference might cause the different result of PslG treatment on the biofilms formed by pathogens from other genera. Nevertheless, minimal media provided similar poor nutrient conditions as wound or skin surface environment. Furthermore, pretreatment with PslG largely enhanced the bactericidal effect of PHMB against *P. aeruginosa* biofilms. The broad function of PslG at eradiating the pathogens biofilms is promising to bring great prospect in new antimicrobial therapy development.

## 4. Materials and Methods

### 4.1. Strains, Media, and Growth Conditions

Four pathogens were tested in this work, including Gram-negative bacteria *Pseudomonas aeruginosa* and *Escherichia coli*, Gram-positive bacteria *Staphylococcus aureus* and pathogenic fugus *Candida albicans* (Table 1). Single colonies were inoculated in the liquid-rich medium and cultured at 200 rpm at 37 ℃. Subsequently, overnight culture was diluted into minimal medium to test the bactericidal and anti-biofilm effect of PHMB (VANTOCIL TG 45014-3628Z, LONZA, West Yorkshire, UK) and UB (REWOTERIC AM B U 185. EVONIK industries, Essen, Germany), since the tryptone and peptone in rich media would form flocculation with the presence of PHMB. Media used for culturing each pathogen were listed in Table 1.

### 4.2. Growth Curves

To determine the growth of pathogens, each strain was inoculated in 96-well plate (701001, NEST Biotech Co., Ltd, Wuxi, China) using a 1:100 dilution inoculum of overnight culture. Ninety-six-well plates were shaken in a microplate incubator (ST60-4, MIULAB, Hangzhou, CHINA) 700 rpm at 30 °C and cell growth was recorded by monitoring OD_600_ every two hours using an automated microplate reader (MULTISKAN-MK3, THERMO Scientific, Waltham, MA, USA).

### 4.3. Initial Attachment Assay

Pathogens overnight culture in rich medium was diluted at 1:100 to the corresponding minimal medium, and then incubated at 37 °C with shaking at 200 rpm to reach OD_600_ at 0.5. One-hundred-microliter of each fresh culture was inoculated into 96-well PVC microtiter dishes (COSTAR-2797, Corning Inc. Kennebunk, ME, USA) and incubated at 30 °C for 30 min (for *P. aeruginosa* attachment) or 1h (for *E. coli*, *S. aureus*, and *C. albicans* attachment), under static stations. PHMB and UB was supplied into the growth media during inoculation as needed. Crystal violet staining assay was performed to determine the microbial attachment as previously reported [46]. Briefly, liquid culture was discarded from the microtiter dishes. Each well was washed and then stained with 120 μL of 0.1% crystal violet (CV, sigma) at 30 °C for approximately 30 min, and then rinsed thoroughly with water for three times. The CV bound to attached microbial cells was subsequently solubilized with 200 μL of 40% acetic acid and measured for absorbance at 560 nm using a microplate reader (MULTISKAN-MK3, THERMO Scientific, Waltham, MA, USA). Each test was performed at least in triplicates. Two tailed *t*-test was performed to discern differences between groups.

### 4.4. Biofilm Formation Assay

Four pathogens were grown in minimal media for biofilm formation assay. The indicated minimal medium was inoculated into 96-well PVC microtiter with a 1:100 dilution from an overnight rich medium culture. PHMB, UB, and PHMB + UB were added at the beginning of inoculation accordingly. Plates were incubated at 30 °C for 6 h, 12 h (only for *P. aeruginosa*), 24 h, and 48 h (except for *P. aeruginosa*). Liquid culture was removed, and wells were washed with ddH_2_O for three times. One-hundred-and-twenty-microliter of a 0.1% solution of CV was added to each well, the plates were incubated at 30 °C for approximately 30 min. CV was dissolved with 40% acetic acid and the absorbance at 560 nm was measured using a microplate reader (MULTISKAN-MK3, THERMO Scientific, Waltham, MA, USA) for the formation of biofilms.

### 4.5. Disruption of Preformed Biofilms

This principle of this assay is similar to biofilm formation and is aimed to disrupt the preformed biofilms. The minimal medium (100 μL/well) for each pathogen was inoculated from a 1:100 dilution from an overnight medium culture. Plates were incubated at 30 °C for 24 h (for *P. aeruginosa*) or 48 h (for the other three pathogens). Then the liquid cultures were replaced by minimal media containing PHMB, UB, PslG [12], PHMB+UB, or PslG+PHMB, respectively, and incubated at 30 °C for 30 min (PslG treatment), and 2 h, 6 h, or 24 h for PHMB and/or UB treatment accordingly. For untreated control wells, the culture was replaced by minimal medium without any antiseptic. After treatment, the liquid culture was pipetted out from each well to determine OD_600_ of planktonic bacteria under a microplate reader (MULTISKAN-MK3, THERMO Scientific, Waltham, MA, USA) as required. The remaining biofilm biomass was determined by CV staining assay as described in the biofilm formation assay.

### 4.6. Image Acquisition and Analysis of Air-Liquid Interface Biofilms (Pellicles)

The air-liquid interface biofilms, termed as pellicles, were grown in four-well glass chambers with individual chamber dimension of 1 × 1 × 4 cm (Lab-Tek II, Chambered # 1.5 German Coverglass system-155382, Thermo Fisher Scientific, Rochester, NY, USA) as described previously [47]. For confocal laser scanning microscopy (CLSM) observation, buffer was gently removed from glass chambers to allow the pellicles to drop onto coverslips. The biofilms were stained with DNA stain SYTO 9, and the membrane-compromised bacteria (consider to be dead cell) were stained with propidium iodide (Molecular Probes, Invitrogen, Eugene, OR, USA). The fluorescent and differential interference contrast (DIC) images were acquired with a FV-1000 confocal laser scanning microscope (Olympus, Tokyo, Japan). The biofilm biomass was quantified by COMSTAT software [48].

### 4.7. Statistics

All experiments were conducted in triplicates. Data were compared by Student’s *t*-test using GraphPad Prism 5 software. *p* < 0.05 was considered statistically significant.

## Figures and Tables

**Table 1:** A list of strains and media used in this study.

Strains	Rich Medium	Minimal Medium *
*Pseudomonas aeruginosa* PAO1 [37]	LBNS medium [38]	Jensen’s medium [39]
*Escherichia coli* JM109 [40]	LB medium [41]	M9 medium [42]
*Staphylococcus aureus* ATCC6538	TSB medium [43]	Jensen’s medium
*Candida albicans* SC5314 [44]	YPD medium [45]	RPMI 1640 medium (Gibco, Life technologies, New York, NY, USA)

* Minimal medium was used in microbial attachment, biofilm formation and all the experiments regarding antiseptics treatment.

## Data Availability

Not applicable.
